# Impact of Substance Use Disorder on Tryptophan Metabolism Through the Kynurenine Pathway: A Narrative Review

**DOI:** 10.3390/metabo14110611

**Published:** 2024-11-10

**Authors:** Lindsey Contella, Christopher L. Farrell, Luigi Boccuto, Alain H. Litwin, Marion L. Snyder

**Affiliations:** 1 Healthcare Genetics and Genomics, School of Nursing, Clemson University, 605 Grove Rd., Greenville, SC 29605, USA; 2 Luxor Scientific, LLC, 1327 Miller Rd., Greenville, SC 29607, USA; 3 School of Health Research, Clemson University, Clemson, SC 29631, USA; 4 Department of Medicine, Prisma Health, 701 Grove Rd., Greenville, SC 29605, USA; 5 Department of Medicine, School of Medicine, University of South Carolina, 876 W Faris Rd., Greenville, SC 29605, USA

**Keywords:** kynurenine pathway, tryptophan, substance use disorder

## Abstract

**Background/Objectives**: Substance use disorder is a crisis impacting many people in the United States. This review aimed to identify the effect addictive substances have on the kynurenine pathway. Tryptophan is an essential amino acid metabolized by the serotonin and kynurenine pathways. The metabolites of these pathways play a role in the biological reward system. Addictive substances have been shown to cause imbalances in the ratios of these metabolites. With current treatment and therapeutic options being suboptimal, identifying biochemical mechanisms that are impacted during the use of addictive substances can provide alternative options for treatment or drug discovery. **Methods**: A systematic literature search was conducted to identify studies evaluating the relationship between substance use disorder and tryptophan metabolism through the kynurenine pathway. A total of 32 articles meeting eligibility criteria were used to review the relationship between the kynurenine pathway, tryptophan breakdown, and addictive substances. **Results:** The use of addictive substances dysregulates tryptophan metabolism and kynurenine metabolite concentrations. This imbalance directly affects the dopamine reward system and is thought to promote continued substance use. **Conclusions:** Further studies are needed to fully evaluate the metabolites of the kynurenine pathway, along with other options for treatment to repair the metabolite imbalance. Several possible therapeutics have been identified; drugs that restore homeostasis, such as Ro 61-8048 and natural products like *Tinospora cordifolia* or *Decaisnea insignis*, are promising options for the treatment of substance use disorder.

## 1. Introduction

Substance use disorder (SUD) is a complex healthcare issue plaguing the United States. SUD is defined by the American Society of Addictive Medicine (ASAM) as a “primary, chronic disease of brain reward, motivation, memory, and related circuitry” [1]. In 2020, 21% of people over the age of 12 in the United States misused prescription or illegal drugs within the previous year [2]. These substances include alcohol, opiates, stimulants, marijuana, and hallucinogens, among others [3]. SUD has been shown to involve complex interactions of genetic makeup, environmental inputs, and brain circuit function [4].

Since the 1960s, it has been hypothesized that SUD has an underlying biological mechanism. While conducting clinical trials for the treatment of individuals using heroin, Marie Nyswander and Vincent Dole proposed that individuals using heroin had undergone specific biological changes compelling continued use [5]. To best treat heroin use disorder, they discovered that the use of methadone allowed patients going through opioid withdrawal to return to their everyday lives. Methadone for SUD restores patient homeostasis, similar to insulin in a person with diabetes [5]. This finding highlighted the need to identify the underlying metabolic changes that occur in substance users to determine alternative drug targets and therapeutic alternatives.

Drugs and alcohol can seize the pleasure/reward circuits in the brain and encourage the user to continue using addictive substances. Biomarkers have been previously identified concerning SUD, including dopamine, serotonin, norepinephrine, endocannabinoids, glutamate, and GABA [6]. Determination of the biochemicals altered during substance abuse can provide evidence to identify alternative treatment and management plans.

### 1.1. Tryptophan Metabolism

**Figure 1:**
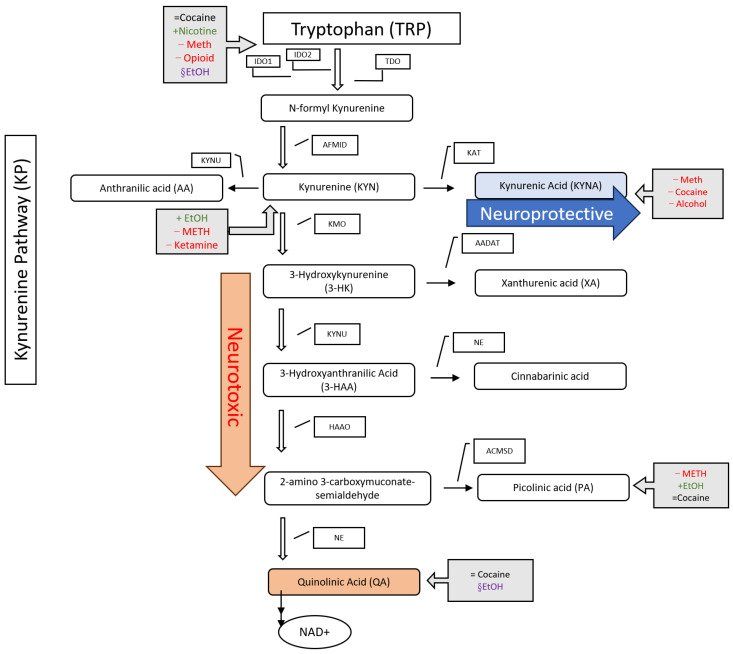
Metabolism of TRP by the KP. The KP has two main branches: the neurotoxic pathway and the neuroprotective pathway. The impacts addictive substances have on KP metabolites in the blood are shown in grey boxes: + indicates an increase, − decrease, = no change, and § mixed results. Enzymes involved in the pathway are shown in shaded boxes. Abbreviations: EtOH: alcohol; METH: methamphetamine; TRP: tryptophan hydroxylase; AA: aromatic acid; IDO1: indoleamine 2,3-dioxygenase-1; IDO2: indoleamine 2,3-dioxygenase-2; TDO: tryptophan 2,3-dioxygenase; KATs: kynurenine aminotransferases; KMO: kynurenine 3-monooxygenase; KYNU: kynureninase; NE: Nonenzymatic; HAAO: 3-hydroxy anthranilate 3,4-dioxygenase; AFMID: arylformamidase; ACMSD: α-amino-β-carboxymuconate-ε-semialdehyde-decarboxylase; AADAT: aminoadipate aminotransferase; NAD+: nicotinamide adenine dinucleotide.

Tryptophan (TRP) is an essential amino acid that has been studied in correlation with substance use disorder [7]. TRP is metabolized by two pathways: the serotonin pathway (SP), which has been known to play a role in SUD, and the kynurenine pathway (KP), which has recently been highlighted for its role in SUD (Figure 1) [7]. While the SP has been extensively studied in individuals with SUD, the KP is less studied regarding SUD, even though it accounts for 95% of the tryptophan pathway compared to the serotonin pathway with 5% [7]. Serotonin is one of the most well-known metabolites of TRP and has previously been identified as a neurotransmitter associated with SUD [8]. The metabolites of these pathways are essential in regulating multiple biological processes, including protein synthesis, neurological functions related to the reward system, and gut microbiota [9]. Melatonin, one of the metabolites of the SP, is essential for sleep quality [10]. The KP creates metabolites resulting in nicotinamide adenine dinucleotide (NAD+), essential for producing and transferring cellular energy. The metabolic pathway leading to the production of serotonin and melatonin, along with broader metabolic interactions such as redox signaling and mTOR activation, profoundly influences mood and behavior and has been linked to numerous neuropsychiatric conditions with noticeable overlaps with SUD. It will also compete with the KP for the utilization of the available TRP in certain tissues and, therefore, may exert indirect regulation of the KP activity as well. However, the scope of this review is exclusively focused on the relationship between SUD and the KP products, so we elected not to explore the role of other pathways, albeit related to TRP. 

As shown in Figure 1, there are two distinct branches in the breakdown of TRP by KP, one leading to Kynurenic acid (KYNA), a neuroprotective product, and the second a quinolinic acid (QA), a neurotoxic product [11]. It has been observed that KYNA (neuroprotective) can bind dopamine/glutamate receptors and block dopamine release in the brain [7]. The neurotoxic metabolite QA is also implicated in mitochondria homeostasis and programmed cellular apoptosis, two critical biological processes [12]. Since metabolites of both branches play critical roles in cellular processes, the metabolite concentrations of the neuroprotective and neurotoxic pathways of the KP need to remain balanced [7]. Although the conversion of TRP metabolism is well understood, ongoing research is determining how substance abuse can cause imbalances in these KP targets, promoting continued use of a given substance and increasing the chance of relapse.

### 1.2. Mechanism of Action

The KP plays a critical role in the cellular process and formation of cellular energy [13]. Multiple factors can upregulate the KP, causing an imbalance in TRP and KP metabolites, including stress, inflammation, gut microbiota, and SUD [9,11]. Imbalances in the metabolites in this pathway have been found to play a role in chronic pain, depression, schizophrenia, other psychiatric illnesses, and inflammatory diseases [4].

One hypothesis for the imbalance in KP metabolites attributes it to the upregulation of cytokines. For example, Bravo et al. summarized the impact of psychostimulants on cytokines profiles, identifying that cocaine and methamphetamine (meth) use upregulate multiple cytokines, including interleukin 6 (IL-6) and IL-1β [14]. These directly impact the KP regulation. These cytokines promote metabolism through the neurotoxic path of the KP by upregulating IDO1/2 and KMO enzymes while inhibiting KAT [15]. This results in an imbalance of the metabolites of the KP, impacting the reward circuit and temporarily increasing dopamine production [7]. TRP metabolism is dysregulated in SUD because addictive substances like alcohol, cocaine, and opioids can dysregulate immune responses and trigger oxidative stress, which shifts tryptophan away from serotonin production and toward kynurenine metabolites. This imbalance impacts neurotransmission, neuroinflammation, and neurotoxicity, contributing to addictive behaviors by affecting pathways like dopamine, thus reinforcing the cycle of SUD [7].

This narrative review focuses on the influence addictive substances have on TRP metabolism through the KP, identifying variations seen between sample types and genders and discussing the current understanding of options for restoring the balance of the metabolites of the KP. A narrative review was chosen to summarize what is known about the dysregulation of the KP following the use of addictive substances to identify overlapping trends and determine areas where further research is necessary. The findings from this review can be used as an aid in identifying novel drug mechanisms and treatment therapies in substance abuse management.

## 2. Materials and Methods

This narrative literature review summarizes published studies examining the influence addictive substances have on TRP metabolism through the KP. The literature search identified clinical studies and animal models that evaluated the role KP plays in substance use disorder.

### Search Strategy and Article Analysis

**Figure 2:**
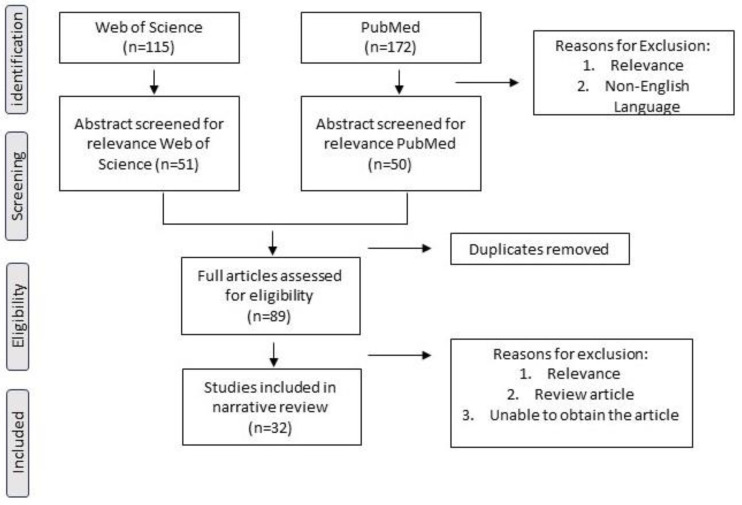
Flow diagram depicting selection of literature included in systematic search using Preferred Reporting Items for Systematic Reviews and Meta-Analyses (PRISMA).

A systematic search was conducted on the Web of Science and PubMed using a combination of search terms relating to TRP, KP, and SUD (listed in Supplementary Table S1). The results were limited to the previous ten years (2013–2023) to highlight the most current understanding of the relationship between TRP breakdown, KP, and SUD. Figure 2 shows the flowchart depicting the article selection. Web of Science yielded 115 articles, and PubMed yielded 172 articles. After article evaluation, 32 were selected for the narrative review based on relevance and availability. Following STROBE (strengthening reporting of observational studies in epidemiology) guidelines, the studies are summarized in Supplementary Table S2. Additionally, the theoretical framework, primary findings, and limitations were analyzed as part of the review process.

## 3. Results

The systematic search produced 32 articles that explore the relationship between substance abuse, TRP breakdown, and KP. A summary of the studies is presented in Table 1. Of the studies, 15 were based on animal models and 17 on human subjects. Eight TRP metabolites were evaluated: 3-hydroxykynurenine (3-HK), anthranilic acid (AA), KYN, KYNA, picolinic acid (PA), QA, TRP, and xanthurenic acid (XA). Of these, TRP, KYN, and KYNA were the most studied. Five sample types, blood, urine, liver, brain, and cecal, were analyzed, assessing SUD’s impact on the concentration of TRP metabolites. Lastly, eight addictive substances, alcohol, methamphetamine, cocaine, heroin, nicotine, ketamine, morphine and THC, have been studied and were identified in the literature as impacting the KP. Alcohol was the most researched analyte in determining its relationship to KP. These articles represent multiple species, drugs, and sample types to further the understanding of the impact addictive substances have on the metabolism of TRP and KP.

This literature review also identified multiple studies that measured the efficacy of treatment options, including probiotics, anti-inflammatories, and enzyme inhibitors, to determine if impacting TRP metabolism can restore homeostasis within the KP. Of these studies that evaluated treatment options, most measured concentration changes in the TRP metabolites. However, two studies used behavioral traits to measure the efficacy of treatment options targeting the KP.

### 3.1. Concentration Variations of TRP and KP Metabolites in SUD

Each substance of abuse can result in an increase, decrease, or null concentration change in the metabolites of KP (Table 2). Identifying the concentration variations in the KP can better identify options for restoring homeostasis or potential drug targets [16]. Different substances enact varying effects on the body, consistent with the idea that they will also impact the KP differently. Table 2 summarizes each drug’s impact on the individual metabolites based on evidence identified in the literature search.

### 3.2. Alcohol

The relationship between alcohol use disorder (AUD), TRP, and KP has been studied extensively. Nineteen articles correlating alcohol consumption with changes in metabolite concentrations were identified in the search, of which nine evaluated the metabolites in animal models [17,18,19,20,21,22,23,24,25] and ten in human samples [26,27,28,29,30,31,32,33,34,35]. TRP and KP metabolites were assessed in five sample types: blood [17,25,27,28,29,30,31,32,33], brain [17,18,21,22,23,24,31,35], liver [20], urine [26,31,34] and cecal [19]. Following alcohol consumption, KYN concentrations generally increased in blood compared to the control group, while KYNA decreased [27,28,29,30,32,33]. Six studies monitored metabolite concentrations in brain samples from rat and mice subjects; when compared to the control group, one study found TRP increased, another study found TRP decreased, two studies found an increase in KYN, two studies found no change in KYN, and two studies found an increase in QA [18,21,22,23,24,35]. Bano et al. compared QA concentrations in brain and liver samples, finding significant changes in the liver and no significant concentration changes in the brain [20]. One study measured TRP and KYN concentrations in cecal samples, finding no significant changes in TRP, but KYN decreased significantly compared to the control group [19]. Although the results were variable in these animal studies, the overall findings are consistent with the hypothesis of an increase in the neurotoxic pathway in SUD, as evidenced by the decrease in neuroprotective KYNA and the increase of neurotoxic QA in blood.

A similar metabolic pattern was observed in humans compared to the animal models; six studies using blood showed an increase in KYN, and four studies found a decrease in KYNA concentration compared to the control group. Leclerq et al. also found that neurotoxic metabolites PA and QA were increased in the alcohol cohort, while the neuroprotective KYNA and AA, 3-HK, and XA decreased [28]. These changes in the blood were not seen across all sample types. Maciejczyk et al. performed a retrospective study of postmortem blood, brain, and urine samples taken during autopsies from alcohol-related deaths [31]. TRP and KYN concentrations were analyzed, and the only significant concentration change observed was an increase in TRP in urine samples [31]. The disagreement between Maciejczyk and the previous results may be attributed to the biochemical processes that occur during and after death and the time from the time of death to the collection of postmortem samples.

Six studies evaluated treatment options targeting the KP in treating AUD. Pizarro et al. evaluated whether synbiotic, a probiotic, in rats with AUD, would restore balance to TRP and KP metabolites and if the results varied between sexes [24]. This study specifically monitored changes in TRP and KYN, alongside the benefits of synbiotics during recovery, using three cohorts, control, alcohol plus water, and alcohol plus synbiotics, and evaluated males and females separately. Their study found that changes in KP metabolites differed between genders. In the female rats, KYN was significantly increased in the alcohol-synbiotic cohort, and no significant change was observed in the water-alcohol cohort compared to the control group. The opposite was observed in the male group; KYN was significantly increased in the water plus alcohol cohort, and no significant change was observed in the synbiotic plus alcohol cohort. TRP concentrations were statistically higher for both genders in the water-alcohol cohort; one difference in TRP concentration was that the female synbiotic-alcohol group was also significantly decreased compared to the control. The differences in metabolic patterns were thought to be attributed to differences in sex hormones between the genders and/or the small sample sizes in the study. Interestingly, synbiotics were shown to reduce relapse likelihood in both genders and can be a treatment option during the critical recovery stages of AUD [24].

Additional studies evaluated anti-inflammatory plants and enzyme inhibitors to determine their impact on recovery. Sharma [26] and Mittal [34] assessed the benefits of the plant *Tinospora cordifolia* (TCE) in reducing toxicity in AUD cohorts. An additional study evaluated *Decaisnea insignis* seed oil in rats with AUD [19]. Two studies assessed the KMO inhibitor, Ro 61-8048 [17,23]. Each study showed that these drugs and supplements decreased relapse in the AUD cohort, presumably by restoring homeostasis within the TRP metabolism and the KP pathway.

### 3.3. Cocaine

Four studies were identified in the literature to determine the impact of cocaine on the KP. Two of the four studies analyzed KP as a target for treatment in reducing the probability of relapse within cocaine-addicted rats and squirrel monkeys [25,36]. Secci [36] and Vengeliene [25] conducted experiments to determine if using Ro 61-8048, an inhibitor of KMO, the enzyme in the reaction producing the neurotoxic product QA, would help reduce the chance of relapse. The results of these experiments showed that pharmacological enhancement of KYNA levels could provide a novel treatment for cocaine use disorder, diminishing the craving and relapse cycle. It is worth noting that similar findings were seen in a study by Justinova et al. that evaluated THC use disorder relapse in rats and squirrel monkeys, and the results correlated with a decrease in relapse in subjects who received the inhibitor [37]. Increasing KYNA is a potential treatment option that warrants further research [16].

A cross-sectional study analyzed 100 subjects with cocaine use disorder to determine if alterations were observed within both the SP and KP [8]. TRP, KYN, KYNA, and QA levels were measured in the blood to determine if a significant difference was observed between cocaine users and the control group. TRP and KYN concentrations did not differ significantly. KYNA was significantly decreased in the cocaine cohort compared to the control; an observed increase in QA was seen but was not statistically significant [8]. This discovery aligns with the potential of Ro 61-8048 to benefit humans, as observed in animal studies, through its ability to increase KYNA concentrations [37]. The findings in this study suggest the existence of clinically relevant biomarkers that could be used for aiding in assessing the severity of SUD in cocaine users and/or used for monitoring the response to treatment.

### 3.4. Methamphetamine (Meth)

The influence of the stimulant meth on the metabolism of TRP has been analyzed in animal models (n = 3) and human subjects (n = 3) [38,39,40,41,42,43]. A study by Kim et al. identified patterns of amino acid metabolism in male rat plasma before and after meth use and in periods of abstinence [40]. Targeted and non-targeted metabolomics was used to determine how abstinence changed concentrations of amino acids and their metabolites. Rat plasma was obtained 16 days after self-administration of meth, along with 12 and 24 h after abstinence. A significant change in TRP was observed across the three collection times [40]. TRP concentration significantly decreased during the initial use of meth compared to the control group; simultaneously, serotonin significantly increased during the use of meth, suggesting TRP was metabolized to serotonin more rapidly than the controls. KP metabolites were not analyzed during the study. TRP concentration was also investigated using a multi-level mixed modeling method in male mice, identifying metabolomic changes in the brain. Unlike in blood, TRP concentrations were found to increase in the brain [38].

Three studies evaluated the impact meth had on TRP concentrations in humans. Wang [43] and Chaidee [41], using cross-sectional studies, found decreased blood TRP concentration. Wang also measured cytokines IL-6 and IL-18, detecting a slight increase in the concentration of IL-6 in the meth group compared to controls [44]. This is consistent with the understanding that IL-6 is upregulated in meth use and can upregulate the conversion of TRP to KYN, consistent with a decrease in the concentration of TRP detected in the blood [14,41]. Meth has been shown to increase inflammatory cytokines IL-6 and IL-18, known to enhance TRP metabolism; these cytokines could work differently in the blood and brain, resulting in varying results [45]. Lastly, Cheng et al. analyzed whether gender affected TRP concentrations following meth use [42]. Within their cohort, the male meth group showed a significant decrease in TRP compared to the control, while the female cohort did not. This outcome suggests that there are sex-specific variations in TRP metabolism during meth use [42].

A review by Davidson [4] discussed the effect meth had on TRP metabolism and the SP and KP pathways. The authors presented that meth also affects TRP metabolism. Their review found that KYN, KYNA, PA, and 5-hydroxy-tryptophan metabolites of the KP and SP were lower in meth users compared to expected concentrations, along with identifying that alcohol, THC, heroin, nicotine, caffeine, and cocaine can also influence metabolites of the SP and KP [4]. This review supported the evidence presented in this literature search that substance use, especially meth, can decrease the generation of neuroprotective metabolites of the KP.

### 3.5. Opioids

The drug class of opioids consists of both prescribed medications and illicit drugs. Commonly abused opioids morphine and heroin were found in the literature to impact TRP metabolism. A limited number of studies have been conducted on the relationship between opioids and KP. Still, TRP concentrations are decreased in heroin and morphine users compared to controls in blood samples [39,46,47]. One study evaluated XA in morphine, and no significant change was observed [47]. Zheng et al. measured TRP concentrations in both urine and blood samples of male rates when given heroin, finding that TRP concentration increased in urine while it decreased in blood [46]. With the increasing opioid crisis in the United States, further research and understanding are needed to determine if the patterns identified in other addictive substances are consistent with opioid use.

### 3.6. Other Addictive Substances

During the literature search, two studies were identified that monitored the relationship of other addictive substances on TRP metabolism. Zhang [48] measured TRP, KYN, and KYNA in chronic ketamine users to determine the role ketamine has on the KP. In a cohort of 78 human subjects, TRP, KYN, and KYNA concentrations were significantly decreased compared to the control group (n = 79). A decrease in the neuroprotective metabolite is consistent with other addictive substances. TRP concentration in nicotine users was evaluated in one study [49]. In this cohort of ninety male dipping tobacco users, TRP concentration was increased compared to the control group (n = 68) [49]. It had previously been determined that IDO1/2 activity is downregulated in nicotine users and could be the cause for an increase in TRP concentration [7].

## 4. Discussion

### 4.1. KP Metabolite Concentrations in SUD

In this literature review, SUD is shown to upregulate the neurotoxic pathway of the KP compared to the neuroprotective pathway. This shift towards the neurotoxic KP pathway is consistent with the understanding that addictive substances have negative impacts on biochemical processes [11]. The most analyzed sample types included blood samples and brain specimens. Brain specimens were analyzed in ten of the identified research studies. Brain specimens are more reflective of the impact of SUD on TRP since most of the metabolism occurs in the brain. Still, it is an invasive sample type and only available in human autopsy cases. The main findings in the brain studies were a decrease in the neuroprotective metabolite KYNA and an increase in the neurotoxic metabolite QA [18]. The variability observed in the results within the brain studies could be attributed to the sampling technique, study design, and the limited number of human brain samples available. Nineteen studies used blood to monitor TRP metabolism’s metabolic patterns, since it typically represents bodily processes. Similar results were obtained in blood studies compared to the brain, with a general decrease in KYNA. In the blood studies, only two tested for QA, and the results were inconsistent [28,29].

Metabolite changes differed in the blood and brain cohort. Blood concentrations of TRP and KP metabolites may not be sufficient in determining variations or abnormalities in TRP and KP metabolites. For example, since IDO and TDO enzymes are not expressed consistently in all cells of the body, and many of these metabolites are unable to cross the blood–brain barrier, changes in metabolites between sample types are expected. Identifying how each sample type concentration is altered can provide insight into the addictive substance mechanism of action and provide awareness of the optimal treatment. Table 2 highlights the concentration changes between the sample types compared to a control group. Identifying the changes caused in blood and urine, which are easier in terms of obtaining sample types, will be necessary to enable KP metabolic profiling to monitor SUD.

Interestingly, gender was shown to play a factor in TRP metabolite concentration. Traditionally, only male rats or mice were used in non-human subject studies, limiting gender as a co-founding variable. A limited number of studies evaluated both genders and found that KP metabolite concentrations differed between the genders. Sex hormones and their physiological impacts were hypothesized to cause the variation seen in the sexes. Differences in gender metabolite profiles will need to be considered with further studies to ensure treatment plans are applicable for each patient with SUD.

### 4.2. Biological Mechanisms Associated with SUD and KP

The use of addictive substances can impact multiple biochemical pathways. One example is the dopamine reward circuitry, which is stimulated with SUD [50]. The current hypothesized theory of SUD and the reward system has multiple stages, starting with occasional-award driven use and ending in persistent use [51]. The first phase is the initial use of a substance and the subsequent release of dopamine, which gives the user a pleasurable response. The next phase is the withdrawal phase; addictive substances alter the dopamine regulation in the brain, and without the drug, the dopamine system is downregulated. This creates the commonly seen negative emotional state associated with withdrawal [50]. The last stage is summarized by intense cravings, typically leading the individual to use the addictive substance and resulting in habitual, compulsive use [51].

SUD and KP metabolites can affect dopamine release. Alcohol, cocaine, meth, nicotine, opioids, and THC have been shown to excite the glutamatergic neuron, which subsequently promotes the release of dopamine in the brain [7]. Metabolites of the KP also affect dopamine release; imbalances in these metabolites can lead to increased dopamine release. KYNA and QA, which were shown to be dysregulated in SUD, directly impact dopamine release. QA, which was found to be increased in SUD, can cause an increase in dopamine, while KYNA, which was found to be decreased, can block dopamine release. This concept points to why one potential therapy, Ro 61-8048, helps modulate the response from addictive substances by increasing the KYNA present in the brain and decreasing relapse probability [16].

Enzymes involved in TRP metabolism are highly impacted by stress and inflammation. As research on the gut biome has evolved, the relationship between the gut biome, KP, and SUD is becoming more understood. The gut microbiota influences many areas of the body, most importantly, the central nervous system (CNS) [52]. The gut microbiota can signal to the CNS via three main pathways: via immune activity and proinflammatory cytokines, through short-chain fatty acids, and by modulating tryptophan metabolism. Abnormalities can impact the signaling of the proinflammatory cytokines and TRP metabolism, ultimately affecting the KP metabolites. As mentioned previously, cytokines, such as IL-6, can upregulate the neurotoxic branch of the KP by promoting and suppressing enzymes [41]. The gut biome can affect the pharmacodynamics and pharmacokinetics of TRP metabolism; SUD alters the gut biome, identifying it as a target for potential therapeutic management.

Most research evaluating SUD and the gut biome was performed in human or animal models with AUD. Engen et al. reviewed alcohol’s impact on the intestinal microbiota composition, finding that alcohol disrupts the microbiota [53]. Studies in rodent models and humans have found that AUD can cause bacterial overgrowth and dysbiosis, an imbalance in bacterial composition, leading to inflammation and altered TRP metabolism [53]. Similar to results observed in the previously stated synbiotic study, both probiotics and prebiotics were shown to improve the gut biome bacteria composition in AUD [24]. Targeting the gut microbiota represents a promising focus in modulating the TRP metabolism, leading to improved treatment therapies in AUD.

### 4.3. Treatment Options to Correct Metabolite Imbalances

Targeting the KP for cognitive and neurodegenerative disorders has been studied [15]. It was also investigated whether regulating the enzymes involved in the pathway is a way to restore homeostasis of the metabolites, specifically IDO, KAT, and KMO, three of the rate-limiting enzymes involved in the pathway [54]. Multiple studies have evaluated if enzyme inhibitors of the KP could be possible treatment options for substance use. For example, Ro 61-8048, an allosteric inhibitor of KMO, has been identified as a potential treatment path for reducing the concentration of neurotoxic metabolites and increasing the neuroprotective metabolites [55]. Inhibiting KMO increases the concentration of KYNA, resulting in a decrease in dopamine release, breaking the cycle of the reward system after drug exposure [7]. Ro 61-8048 was evaluated in five of the articles identified and was shown to decrease relapse of multiple substances (alcohol, cocaine, THC) across several species (rats and squirrel monkeys) [17,23,25,36,37]. These studies showed that pharmacological enrichment of endogenous KYNA concentration provides a novel treatment strategy to interfere with the reward circuit involved with addictive substances as well as with craving and relapse in SUD.

Additional novel treatment strategies for targeting KP metabolites to decrease the chance of relapse have been researched. TCE and *Decaisnea insignis* seed oil were evaluated for their treatment potential in modulating the KP in AUD. TCE has been studied primarily in Indian medicine and has been shown to have anti-toxic and anti-inflammatory properties; *Decaisnea insignis* also has anti-inflammatory properties and can inhibit cytokines like IL-6 [56,57]. Both substances balanced the neurotoxic and neuroprotective pathways, decreasing relapse within these patient cohorts [19,26,34]. As discussed, the regulation of KP is highly dependent on the gut biome, and TCE can increase the intestinal absorption of the vitamins and amino acids seen in deficiencies within AUD cohorts [58]. TCE contains several bioactive compounds, including alkaloids, terpenoids, and phenolic compounds that help reduce inflammation and oxidative stress, which re-establish the balance of TRP, KP, and SP, helping in the recovery of AUD [26]. Christensen et al. conducted a study to determine the role of vitamins in the KP, specifically measuring 3-HA and 3-HK after bariatric surgery when taking a vitamin B regime compared to a control, and positive results were observed [45]. Vitamin B6 could work similarly to Ro 61-8048 by inhibiting the KMO enzyme; unlike Ro 61-8048, it will simultaneously upregulate the KAT enzyme, creating more KYNA [59]. Based on the current evidence in the literature on vitamins’ role in KP, this opening could be further explored regarding SUD [60]. Lastly, TRP is an essential amino acid, which means it is fully obtained from the diet, and altering an individual’s diet to increase the consumption of TRP needs further investigation in SUD. Żarnowska et al. investigated KP metabolite changes when prescribing a ketogenic diet in epilepsy cases [61]. The study showed a statistically significant increase in KYNA concentrations in patients consuming a ketogenic diet compared to the control [61]. This study provides evidence to support that altering diet can be a potential therapeutic option.

This literature search was able to identify the metabolic patterns seen in SUD, providing insight into both the mechanism of action of addictive substances and highlighting potential targets for drug therapies. Summarizing current treatment options that can benefit SUD and AUD patients is essential, as current treatment plans with traditional management are suboptimal.

### 4.4. Limitations

The current research focuses heavily on the impact of alcohol on KP. Still, minimal research has been completed on opioids and cocaine, two highly abused compounds in the US, as well as others. Further studies on these drugs can show if the current understanding of the impact of SUD on KP is transferable to other drugs. Second, TRP is the most analyzed compound, appearing in 25 different studies; KYNA was measured in 11, and QA only in 6. Measuring the full spectrum of metabolites involved in TRP metabolism can provide a further understanding of the role of each addictive substance. Analyzing the concentration of QA can help prove the hypothesis that neurotoxic compounds increase with SUD. Many other factors, including stress, gut biome, inflammation, protein consumption, and hydration, can influence TRP metabolism; a limited number of studies have evaluated these along with the KP metabolites to determine their impact. Future studies could also use biomarkers like cortisol, protein, creatinine, and gut bacteria to decide whether or not they are co-factors. Lastly, gender is an essential factor, but only a few select articles measured variations between gender and its role in metabolic concentrations. These studies identified differing metabolic fingerprints and showed that further research is needed to determine how sex impacts TRP metabolism in SUD. The KP could be a target for treatment, as shown in multiple studies, identifying the need for continued research on the relationship between SUD and KP.

## 5. Conclusions

This narrative literature review evaluates the intricate relationship between the KP and SUD. With the dramatic increases in SUD that the US healthcare system is currently managing, identifying the molecular mechanisms underlying SUD is necessary. Understanding the metabolic pathways impacted during addictive behaviors can help identify targets for drug discovery and inform precision medicine, nutritional intervention treatment and monitoring, and earlier risk detection. One pathway that is affected by SUD is the breakdown of TRP by the KP. In general, neurotoxic KP metabolites are increased with SUD, while the neuroprotective KP is downregulated (Figure 1) [43].

Understanding the molecular mechanism between SUD and TRP metabolism holds promise for developing targeted interventions and therapeutic strategies. The most studied treatment option is Ro 61-8048, a KMO inhibitor. Inhibition of KMO can increase the neuroprotective KYNA metabolite concentrations and help regulate dopamine release in SUD. Other explored treatment options are probiotics and anti-inflammatory medicines, which appeared to offer effective treatments to restore the balance of the metabolites of the KP. Lastly, further research is needed to understand if changes in diet or vitamin B would benefit patients in recovery [11,59,61].

The KP metabolites can provide information on the critical period when the possibility of relapse is the highest, ensuring proper treatment management. Continued research unraveling the complex relationship between SUD and TRP metabolism leading to novel treatment avenues and preventative measures becomes increasingly evident, offering opportunities for more effective approaches to addressing the challenges posed by treating SUD.

## Figures and Tables

**Table 1:** Thirty-two (32) articles were analyzed to determine the relationship between TRP metabolism through the KP and SUD. This table represents the number of articles studying addictive substances, sample types, and metabolites in animal and human models. * Two studies used behavioral traits as variables. EtOH; alcohol or ethanol, THC; tetrahydrocannabinol.

Literature Review Characteristics		Animal	Human	Grand Total
	Total	15	17	32
Addictive Substance	EtOH	9	10	19
	Methamphetamine	3	3	6
	Cocaine	3	1	4
	Heroin	1	1	2
	Nicotine	1	1	2
	Ketamine		1	1
	Morphine	1		1
	THC	1		1
	**Grand Total**	**19**	**17**	**36**
Sample Type	Blood	5	14	19
	Brain	8	2	10
	Urine	2	3	5
	NA *	2		2
	Cecal	1		1
	Liver	1		1
	**Grand Total**	**19**	**19**	**38**
Metabolites	Tryptophan (TRP)	10	15	25
	Kynurenine (KYN)	7	9	16
	Kynurenic Acid (KYNA)	5	6	11
	Quinolinic Acid (QA)	2	4	6
	Xanthurenic Acid (XA)	1	2	3
	Picolinic Acid (PA)		2	2
	3-Hydroxykynurenine (3-HK)		1	1
	Anthranilic Acid (AA)		1	1
	**Grand Total**	**25**	**40**	**65**

**Table 2:** The effect each addictive substance has on TRP and KP metabolites. Each column represents an addictive substance, the rows are each metabolite of the KP, and each box has an arrow signifying up for the increase in metabolite concentration, down for a decrease, and which source and sample type that change was observed in. = No change was observed. § Concentration changes reported did not agree. Red: Neurotoxic; Blue: Neuroprotective. * Two studies used behavioral traits as variables.

	Ethanol	Opioid	Methamphetamine	Cocaine	Nicotine	Ketamine
3-Hydroxykynurenine	↓H,Bl					
Anthranilic Acid	↓H,Bl					
Kynurenic Acid	↓H,Bl/A,Br =A,Bl		↓H,Br	↓H,Bl		↓H,Bl
Kynurenine	↑H,Bl/A,Br =A,Bl/H,Br/U		↓H,Br			↓H,Bl
Picolinic Acid	↑H,Bl↓H,U			=H,Bl		
Quinolinic Acid	§H,Bl ↑A,Br/L			=H,Bl		
Tryptophan	* ↑_H,U_ §_A,Br_ =A,Bl	↑H,U↓H,Bl/A,Bl	↑A,Br↓H,Bl/A,Bl	=H,Bl	↑H,Bl	↓H,Bl
Xanthurenic Acid	↓H,Bl ↑H,U					
	Key					
	H-Human	Bl-Blood				
	A-Animal	Br-Brain				
		L-Liver				
		U-Urine				

## Data Availability

Data sharing does not apply to this article as no new data were created or analyzed in this study.

## Supplementary Materials

The following supporting information can be downloaded at: https://www.mdpi.com/article/10.3390/metabo14110611/s1, Table S1: Search Strategy; Table S2: Literature Search Review Summary.
