# Supplementary material for: Impact of Substance Use Disorder on Tryptophan Metabolism Through the Kynurenine Pathway: A Narrative Review
Source: Metabolites. 2024 Nov 10;14(11):611. doi: 10.3390/metabo14110611 (PMC11597030; doi:10.3390/metabo14110611)
Supplement: Supplementary file 1 [file metabolites-14-00611-s001.zip › metabolites-3247105-supplementary.pdf]

**Supplementary Table S1.** The search strategy deployed for a narrative review on the Web of Science and PubMed.
**Web of Science**
(((TI="kynurenine Pathway" OR AB="kynurenine Pathway") OR (TI=tryptophan OR AB=tryptophan) OR (TI=IDO OR AB=IDO) OR (TI="indoleamine 2,3-dioxygenase" OR AB="indoleamine 2,3-dioxygenase") OR (TI=TDO OR AB=TDO) OR (TI="tryptophan 2,3-dioxygenase" OR AB="tryptophan 2,3- dioxygenase") OR (TI="quinolinic acid" OR AB="quinolinic acid") OR (TI="Xanthurenic acid" OR AB="Xanthurenic acid") OR ALL=Kynurenine))
AND
(((TI="substance abuse disorder" OR AB="substance abuse disorder") OR (TI=addiction OR AB=addiction) OR (TI="opioid use disorder" OR AB="opioid use disorder") OR (TI="opioid abuse" OR AB="opioid abuse") OR (TI="drug abuse" OR AB="drug abuse") OR (TI="substance dependence" OR AB="substance dependence") OR (TI=alcoholism OR AB=alcoholism) OR (TI="substance-related disorder" OR AB="substance-related disorder") OR (TI="drug dependence" OR AB="drug dependence") OR (TI=methamphetamine OR AB=methamphetamine) OR (TI=cocaine OR AB= cocaine) OR ALL="Substance-Related Disorders"))
**PubMed**
(("kynurenine Pathway"[Title/Abstract] OR tryptophan[Title/Abstract] OR IDO[Title/Abstract] OR indoleamine 2,3-dioxygenase[Title/Abstract] OR TDO[Title/Abstract] OR tryptophan 2,3-dioxygenase[Title/Abstract] OR quinolinic acid[Title/Abstract] OR "Xanthurenic acid" [Title/Abstract] OR "Kynurenine"[Mesh]))
AND
(("substance abuse disorder" [Title/Abstract] OR addiction [Title/Abstract] OR "opioid use disorder" [Title/Abstract] OR "opioid abuse" [Title/Abstract] OR "drug abuse" [Title/Abstract] OR "substance dependence" [Title/Abstract] OR alcoholism [Title/Abstract] OR "substance-related disorder" [Title/Abstract] OR "drug dependence" [Title/Abstract] OR "methamphetamine" [Title/Abstract] OR "cocaine" [Title/Abstract] OR "Substance-Related Disorders"[Mesh]))

Supplementary Table S2. Characteristics of articles identified in the literature search.

(Author, Year)	Title	Methodology + Instrumentation	Population	Sample Type	Addictive Substances	Applicable Metabolites	Main Findings
Araos, 2019	Serotonin is the main tryptophan metabolite associated with psychiatric comorbidity in abstinent cocaine-addicted patients	Cross-Sectional Study- ELISA, LC-UV	Human N=160 (cocaine n=100, control n= 60)	Blood	Cocaine	TRP, KYN, KYNA, QA	KYNA significantly lower in cocaine group compared to control. TRP and KYN no significant change seen, QA increased in cocaine withdrawal cohort, but not significantly.
Sharma, 2016	Protective Effects of Tinospora cordifolia on Hepatic and Gastrointestinal Toxicity Induced by Chronic and Moderate Alcoholism	Experiment HPLC-QTOF/MS	Human (Moderate Alcoholic: n=12) Control: n=14	Urine	Alcohol	XA	XA conc. increased with moderate alc use, but stabilized with TCE treatment
Chaidee, 2023	Cognitive impairment and changes of red blood cell components and serum levels of IL-6, IL-18, and L-tryptophan in methamphetamine abusers	Retrospective Study - LC-MS, QTOF	Human N=30 (Meth n=15 (M= 8, F= 7) , HC n=15 (M=6, F=9))	Blood	Meth	TRP	Trp conc. was significantly decreased in Meth abusers compared to control.
Cheng,Z, 2023	Sex-specific metabolic signatures in methamphetamine addicts	Cross-Sectional - UHPLC-MS	Human (Meth: M=17, F=10), (Control: M=20, F=10)	Blood	Meth	TRP	Trp downregulated in male meth users, no change in TRP seen in females. Variations in the metabolic finger print between sexes was observed.
Jang, 2022	The Kynurenine Pathway and Mediating Role of Stress in Addictive Disorders: A Focus on Alcohol Use Disorder and Internet Gaming Disord	Cross-Sectional Study - LC-MS/MS	Human (AUD n=30, Internet Gaming n=34, Controls n=35)	Blood	Alcohol	TRP, KYN, KYNA	TRP conc did not have a significant change, KYN conc increased, and KYNA conc. decreased in AUD compared to controls.
Leclercq, 2021	Alterations of kynurenine pathway in alcohol use disorder and abstinence: a link with gut microbiota, peripheral inflammation and psychological symptoms	Experiment - HPLC-UV	Human (AUD n=57, Control n=16)	Blood	Alcohol	TRP, KYN, KYNA, QA, PA, AA, 3-HK, XA	No significant change in TRP seen. KYNA, AA, 3-HK, and XA conc. dec compared to control. PA and QA conc. incr. compared with control group. KYN was significantly incr. compared to controls at the end of the 18 day detoxification. Monitoring patterns of the KP can detect the critical period when a patient is mostly likely to relapse.

Mechtcheriakov, 2022	Tryptophan-kynurenine metabolism during acute alcohol withdrawal in patients with alcohol use disorder: The role of immune activation	LC-UV, LC-MS/MS, QTOF	Human AUD N=22 (Male n=16, Female n=6)	Blood	Alcohol	TRP, KYN, KYNA, QA	TRP conc. Did not have a significant change between control and samples in acute withdrawal. KYN conc. Inc in each group compared to control. KYNA and QA conc. decreased in AUD group compared to a control group.
Gleissenthall, 2014	Tryptophan metabolism in post-withdrawal alcohol-dependent patients	Experiment - HPLC-UV	Human N=44 (Male n=40, Female n=14)	Blood	Alcohol	TRP, KYN	KYN increased during recovery, possible activation of IDO in early stages of alcohol withdrawal and activation of TDO at the week 8-12 mark.
Maciejczyk, 2022	Do Circulating Redox Biomarkers Have Diagnostic Significance in Alcohol-Intoxicated People?	Retrospective Study - HPLC-UV	Human N=52 (Alcohol: n=22) (Sober: n=30)	Blood, Brain, Urine	Alcohol	TRP, KYN	Trp conc. increased in the urine of the study group in comparison to the control group. No significant change was seen in Kyn conc in any sample type and no change in TRP in blood or brain.
Neupane, 2015	The relationship of alcohol use disorders and depressive symptoms to tryptophan metabolism: cross-sectional data from a Nepalese alcohol treatment sample	Experiment - HPLC-UV	Human, N= 169 (Control Group - N/A)	Blood	Alcohol	TRP, KYN	KYN conc. increased with greater severity of alcohol use disorder. Gender did not impact conc. of KYN or TRP.
Vidal, 2020	Plasma tryptophan and kynurenine pathway metabolites in abstinent patients with alcohol use disorder and high prevalence of psychiatric comorbidity	Experiment- LC-UV	Humans (AUD n=130, Control n= 80)	Blood	Alcohol	TRP, KYN, KYNA	The concentration of metabolites was different between the sexes. Trp conc. was increased in males, but decreased in females compared to control groups. Both sexes had KYN conc increased, and KYNA conc. decreased compared to control group.
Justinova, 2013	Reducing cannabinoid abuse and preventing relapse by enhancing endogenous brain levels of kynurenic acid	Experiment- self-administered model of THC abuse - count levers	Rats and Squirrel Monkeys	NA	THC	KYNA	Enhancing the KYNA levels in the brain reduce cannabinoid use and prevents relapse.
Mittal, 2015	Detection of New Human Metabolic Urinary Markers in Chronic Alcoholism and Their Reversal by Aqueous Extract of *Tinospora cordifolia* Stem	Experiment - HPLC-Q-TOF-MS	Male Human (Alcoholic n =26, Control n = 28)	Urine	Alcohol	PA	PA conc. decreased in the alcoholic group compared to the control. Treatment with *Tinospora cordifolia* (TCE) showed to reverse the impacts of alcohol.
Kim, 2019	Integrated Non-targeted and Targeted Metabolomics Uncovers Dynamic Metabolic Effects during Short-Term Abstinence in Methamphetamine Self-Administering Rats	Experiment- UPCL-QTOF-ESI-MS	Male Rats N=32 (Meth: n =24, Control: n=8)	Blood	Meth	TRP	Trp significantly decreased with self-administration of meth, showing the dynamic metabolic disturbance caused by meth. Glutamate-glutamine homeostatis important pathology of meth addiction.
Kashem, 2016	Metabolomics of Neurotransmitters and Related Metabolites in Post-Mortem Tissue from the Dorsal and Ventral Striatum of Alcoholic Human Brain	Retrospective Study - LC-MS/MS	Male Human (AUD n=6, Control n=6)	Brain	Alcohol	TRP	No significant change was observed in Trp in the regions of the brain (caudate nucleus, putamen, nucleus accumbens) analyzed.

Giménez-Gómez, 2018	Increasing kynurenine brain levels reduces ethanol consumption in mice by inhibiting dopamine release in nucleus accumbens	Experiment - HPLC-UV	Male Mice	Blood, Brain	Alcohol	KYN, KYNA	KYN and KYNA conc. not significantly altered in alchol group compared to water control group. KYN and KYNA conc. Increased significantly with administration Ro 61-8048 reducing chance of relapse.
Jiang, 2020	Indoleamine-2,3-Dioxygenase Mediates Emotional Deficits by the Kynurenine/Tryptophan Pathway in the Ethanol Addiction/Withdrawal Mouse Model	Experiment HPLC	Male Mice N= 400 (Alcohol n= 320 , control = 80)	Brain	Alcohol	TRP, KYN, KYNA, QA	KYN + QA conc. increase was found in the hippocampus, cerebral cortex, and amygdala of ethanol addiction/withdrawal mice. Abnormal expression of KYNA was seen.
McClay, 2013	Large-scale neurochemical metabolomics analysis identifies multiple compounds associated with methamphetamine exposure	Multi-level mixed modeling- LC-MS/MS, UPLC-LTQ, GC-MS	Male Mice N=150	Brain	Meth	TRP	Trp conc. increased with meth administration compared to control
Liu, 2019	Gut Microbiota and Metabolome Response of Decaisnea insignis Seed Oil on Metabolism Disorder Induced by Excess Alcohol Consumption	Experiment- GC-TOF/MS	Male Mice N=50 (Alcohol w/ DISO: n = 40, Control: n =10)	Cecal	Alcohol	TRP, KYN	Kyn conc. decreased in alcohol group compared to control group who were just administered alc. Trp conc. Did not show a significant change, but the study showed Trp metabolism plays a significant role in metabolic disorder with alcohol and during modulation with *Decaisnea insignis* Seed Oil.
Bano, 2019	Increase In Hepatic Quinolinic Acid Concentrations In Alcohol Withdrawn Rats	Experiment HPLC	Male Rats N=30, n=6 for each each group (chow control, liquid diet, maltose dextrin, 8% ethanol diet, alchohol withdrawal)	Brain, Liver	Alcohol	TRP, QA	TRP conc. was measured in the brain, increase seen in ethanol group and no significant change in alcohol withdrawal group compared to control. QA conc. increased in ethanol and ethanol withdrawal group compared to control in liver, no significant change was seen in brain QA conc. 5HT a metabolite of the serotonin pathway significantly increased during ethanol group and significantly decreased in withdrawal group.
Dos Santos, 2021	Early and late behavioral consequences of ethanol withdrawal: focus on brain indoleamine 2,3 dioxygenase activity	Experiment - HPLC-UV	Male Rats N=53	Brain	Alcohol	TRP, KYN	KYN conc. were significantly increased in pre frontal cortex samples in the 21-day withdrawal cohort compared to the control. The ratio ofKYN/TRP inc compared to control, showing an increase in conversion of TRP to KYN.
Gushcha, 2019	Neurotransmitter Disturbances in Some Parts of the Rat Brain and their Correction Under Chronic and Intermittent Alcohol Intoxication	Experiment - HPLC-UV	Male Rats N=59	Brain	Alcohol	TRP	Trp conc. increased with chronic and intermittent alcohol intoxication in the midbrain brain of rats. No difference in TRP was observed in the cerebellum of rats. Utilizing the amino acid Titacin helped

							regulate the conc and could serve as a potential treatment for alcoholism.
Sajid, 2017	Increased HPA Axis Activity and Serum Tryptophan in Naswar (Dipping Tobacco) Users: A Case-Control Study	Experiment - ELISA and Spectroflourimetric	Male Human (Naswar Users n=90, Controls n=68)	Blood	Nicotine	TRP	Trp + Cortisol conc. increased compared to control groups.
Secci, 2017	Attenuating Nicotine Reinforcement and Relapse by Enhancing Endogenous Brain Levels of Kynurenic Acid in Rats and Squirrel Monkeys	Experiment- reward and relapse with nicotine and cocaine	Rats and Squirrel Monkeys	NA	Nicotine, Cocaine	NA	These findings suggest that KMO inhibition may be a promising new approach for the treatment of nicotine addiction.
de Biedma-Elduayen, 2022	Influx of kynurenine into the brain is involved in the reduction of ethanol consumption induced by Ro 61-8048 after chronic intermittent ethanol in mice	Cross-Sectional Study-<br>LC-UV	Mice N=384 (Male n=341, Female n=45)	Brain	Alcohol	KYN, KYNA	KMO inhibition using Ro-61-8048 decreases consumption of and preference for ethanol in the CIE model, Ro greatly increased KYN concentrations in both plasma and limbic forebrain. No comparison between healthy control and alcohol use concentration was made.
Pizarro, 2021	Sex-Specific Effects of Synbiotic Exposure in Mice on Addictive-Like Behavioral Alterations Induced by Chronic Alcohol Intake Are Associated With Changes in Specific Gut Bacterial Taxa and Brain Tryptophan Metabolism	Experiment- HPLC-MS/MS	Rats - N=48 (Alcohol: Female (n=8) + Male n=10) (Alcohol + Synbiotic: Female n=8, Male n=10) (Contrl: Female n=6, Male n=6)	Brain	Alcohol	TRP, KYN	Trp conc. decreased in Alc and Alc + syn. Kyn conc. Females - increase in alc + syn compared to control, Males - increase in alc+water compared to control Synbiotic treatment reduced escalation and relapse to alcohol intake in females and males.
Vengeliene, 2016	Metabolic shift of the kynurenine pathway impairs alcohol and cocaine seeking and relapse	Experiment- ELISA	Rats N=109	Blood	Alcohol, Cocaine	KYN, KYNA	Pharmacological enhancement of endogenous kynurenic acid levels provides a novel treatment strategy to interfere with glutamatergic/NMDAR activity as well as with craving and relapse in alcohol-dependent and substance use disorder patients.

Wang, 2021	Untargeted metabolomics analysis by gas chromatography/time-of-flight mass spectrometry of human serum from methamphetamine abusers	Cross-Sectional Study- GC-TOF/MS	Human (Meth Users n=80 (M=61, F=19), Controls n=80 (M=58, F=22)	Blood	Meth	TRP	Trp conc. decreased in the meth user group compared to control, demonstrating meth abuse contributes to the disturbance of metabolic homeostatis.
Zaitsu, 2014	Metabolic profiling of urine and blood plasma in rat models of drug addiction on the basis of morphine, methamphetamine, and cocaine-induced conditioned place preference	Experiment- GC-MS	Male Rats N=24	Blood, Urine	Cocaine, Meth, Morphine	TRP, XA	Trp conc. decreased in plasma of the morphine group. XA conc. results not reported. Metabolomics could be used to predict the degree of drug addiction.
Zhang, 2019	Preliminary comparative analysis of kynurenine pathway metabolites in chronic ketamine users, schizophrenic patients, and healthy controls	Experiment - LC-MS/MS	Human (Ketamine n=78, Contols n=79)	Blood	Ketamine	TRP, KYN, KYNA	Trp, KYNA, KYN conc. was decreased in chronic ketamine users
Zheng, 2013	Metabolic phenotype of rats exposed to heroin and potential markers of heroin abuse	Experiment - GC-TOF/MS	Male Rats	Blood, Urine	Heroin	TRP	Trp conc. increased in urine but decreased in blood samples in the heroin group compared to the control. Heroin withdrawal of 4 days restored Trp concentration to baseline.
Zhou, 2021	Plasma metabolites changes in male heroin addicts during acute and protracted withdrawal	Cross-Sectional Study - UPLC-MS/MS	Male Human (Heroin: n=35, Control n=15)	Blood	Heroin	TRP	Trp conc. dec. in plasma samples of acute and protracted withdrawal of heroin cohort compared to controls.
